# Phosphorylation promotes the endonuclease-like activity of human centrin 2†

**DOI:** 10.1039/d2ra03402f

**Published:** 2022-08-09

**Authors:** Jing Yang, Yaqin Zhao, Binsheng Yang

**Affiliations:** Institute of Molecular Science, Key Laboratory of Chemical Biology of Molecular, Shanxi University Taiyuan 030006 China yangbs@sxu.edu.cn +86 351 7016358

## Abstract

Centrin is a member of the EF-hand superfamily of calcium-binding proteins, which is involved in the nucleotide excision repair (NER). Reversible phosphorylation of centrin is an important regulatory mechanism *in vivo* and is closely related to many physiological processes. To explore the possible role of centrin in NER, the endonuclease-like activity of human centrin 2 (HsCen2) regulated by phosphorylation in the absence or presence of Tb^3+^ was investigated by spectroscopy techniques, gel electrophoresis, and molecular docking simulation in 10 mM Hepes, pH 7.4. The results showed that phosphorylation weakened the binding of Tb^3+^ to HsCen2 and enhanced the binding of DNA to HsCen2. Phosphorylation improves the endonuclease-like activity of HsCen2. In addition, Tb^3+^ is favorable for DNA binding and endonuclease-like activity of HsCen2 before and after phosphorylation. These results provide clear insights into the effects of phosphorylation on the properties of HsCen2 and offer important clues for further exploration of how phosphorylation affects protein-driven functions.

## Abstract 2

Phosphorylation weakened the binding of Tb^3+^ to HsCen2, enhanced the binding of DNA to HsCen2; and improves the endonuclease-like activity of HsCen2; Additionally, the endonuclease-like activity of HsCen2 or HsCen2p is regulated up by Tb^3+^-binding.

## Graphical abstract

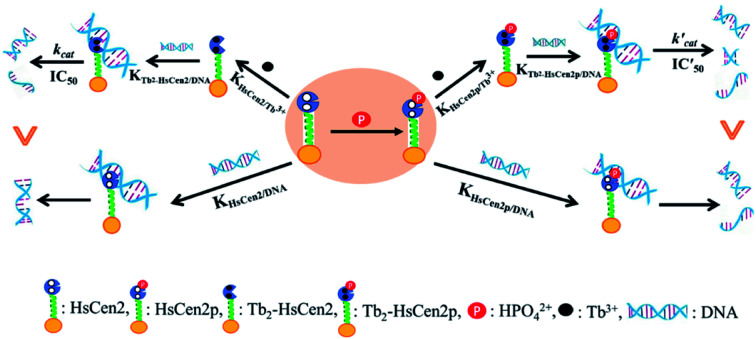

## 1 Introduction

Centrin was first identified in unicellular green algae, such as *Tetraselmis* striata and *Chlamydomonas* reinhardtii, as major components of several types of basal body associated and Ca^2+^-sensitive contracting fibers.^1–3^ It is a relatively small molecular mass (about 20 kDa), acidic, and highly conserved Ca^2+^-binding protein belonging to the EF-hand superfamily.^4^ Generally, centrin contains four helix-loop-helix topology structures, the so-called EF-hand subdomain.^5^ The EF-hand structural motif was devised in 1973.^6^ It is the most common Ca^2+^ binding motif found in proteins.^6^ Centrin is a typical calcium-binding protein. Each domain of centrin has two potential Ca^2+^ binding sites. However, four EF-hand structural primitives of centrin have different affinities for Ca^2+^.^7–10^ The binding of Ca^2+^ results in a major transformation of the centrin conformation from a “closed” to an “open” state.^11^ Some non-polar amino acid residues are exposed to form a hydrophobic pocket, which provides a good condition for the interaction between the protein and downstream target protein to achieve its biological function. At the same time, the Ca^2+^ signal is transmitted through the conformational transformation of proteins.^12^ Centrin is mainly concentrated around microtubule organizing centers (MTOCs) and is closely related to centrosomes and matrix functions.^13^ Centrin is not only located in the centrosome but also distributed in the nucleus or cell matrix.^14^ Centrin is also primarily involved in other cellular processes, including participation in nucleotide excision repair (NER), paramecium ciliary Ca^2+^ channel voltage-gated activity, light transmission of light receptor cells, and the nuclear mRNA export machinery.^15^ Human centrins comprise four different types, namely centrin 1, centrin 2, centrin 3 and centrin 4 (abbreviation: HsCen1, HsCen2, HsCen3, and HsCen4, respectively). HsCen2 and HsCen3 are generally expressed in human cells. HsCen1 is only located in male germ cells, neurons, and ciliary cells. HsCen4 is found in human brain neurons.^16,17^

HsCen2 has 172 amino acid residues and is without tryptophan (Trp) or cysteine (Cys). The only tyrosine (Tyr) is located at the end of the C-terminal of HsCen2. It contains four EF-hand domains. However, there are only two EF-hand domains localized at the C-terminal domain to bind the metal ion (PDB 2GGM).^18,19^ Biochemical and biophysical studies suggest that the centrin and its downstream target proteins jointly participate in the process of nucleotide recognition, resection, and repair, among which xeroderma pigmentosum complement protein C (XPC) is a critical target protein of centrin.^20–27^ The XPC–hHR23B–HsCen2 heterotrimer performs the task of DNA damage identification in global gene NER,^22^ thereby recruiting downstream proteins to initiate nucleotide resection and repair process. In addition, it also found some evidence of direct contact between HsCen2 and DNA.^28^

Protein reversible phosphorylation is a part of the most common post-translational modifications (PTMs) in eukaryotic proteins. It is the most common and main mechanism for regulating cell signal transduction, protein activity, and function.^29^ About 30% of proteins in eukaryotes are phosphorylated in the body.^30^ In the process of phosphorylation regulation, cell morphology and functions are changed.^31^ Reversible phosphorylation involves almost all physiological and pathological processes, including the cell cycle, regulation of gene transcription and DNA replication, metabolic pathways, neuron growth, signal transduction, and memory processes.^32^ Both human centrin and green algae centrin can be phosphorylated *in vivo*.^33–35^ Under normal circumstances, molecular modifications and interactions, including phosphorylation or dephosphorylation, play a very precise role in regulating the centrosome cycle and overall cell proliferation. Once this balance is disrupted, cells may become cancerous or die.^36^ Studies show that many human diseases are closely related to abnormal protein phosphorylation, such as cancer,^37^ diabetes,^38^ and Alzheimer's.^39^ Therefore, it is necessary to study the effect of phosphorylation on the function of proteins. The HsCen2 phosphorylation is related to the replication of the centrosome.^40,41^ Our previous research proves that the interaction between HsCen2 and XPC peptide is double-regulated by phosphorylation and Ca^2+^.^34^

As terbium ions (Tb^3+^) have similar chemical hardness, ion radius, coordination number, and electrostatic coordination characteristics to Ca^2+^,^42,43^ Tb^3+^ has been widely used as a fluorescent probe to study Ca^2+^-binding proteins.

Indeed, phosphorylation plays a very important regulatory role in the functions of HsCen2. The functions of HsCen2 often depend on the participation of the cooperator, such as metal ions and DNA.^44^ However, the functions of HsCen2 after phosphorylation have been less studied. In the study, the effects of phosphorylation on the binding of Tb^3+^ to HsCen2, binding of DNA to HsCen2, and endonuclease-like activity of HsCen2 in the absence or presence of Tb^3+^ are investigated using spectroscopy techniques, gel electrophoresis methods, and molecular docking simulation in 10 mM Hepes, pH 7.4. This work is beneficial for further understanding the effect of phosphorylation regulations or mechanisms.

## 2 Experimental

### 2.1 Reagents

4-(2-Hydroxyethyl)-1-piperazineethanesulfonic acid (Hepes), ampicillin (Amp), and isopropyl-β-d-thiogalactopyranoside (IPTG) were purchased from Sangon in Shanghai of China. Adenosine triphosphate disodium salt (ATPNa_2_) was purchased from Solarbio Corporation. Tb^3+^ solution was obtained from Tb_4_O_7_. Tb_4_O_7_ was dissolved in concentrated hydrochloric acid and using xylenol orange as an indicator, the solution was titrated with standard EDTA complexation in HAc/NaAc buffer at pH 5.7. Protein kinase A (PKA) is self-purified.^43^ DNA (pBR322 DNA, calf thymus DNA) was purchased from Sigma. Biochemical reagents in the construction, expression, and purification of proteins were obtained from Trans Gene. Other chemicals were of the highest purity available from local sources.

### 2.2 Preparation of DNA

The stock solution of calf thymus DNA (CT-DNA) was prepared in 10 mM Hepes, pH 7.4. The concentration of DNA was determined by the absorbance at 260 nm using a molar extinction coefficient of *ε*_260_ = 6600 M^−1^ cm^−1^. The DNA solution exhibited an ultraviolet absorbance ratio (A260/A280) of 1.89, demonstrating that DNA was sufficiently free from protein.^45^ The DNA solution used in all experiments was CT-DNA, except for that in agarose gel electrophoresis experiments.

### 2.3 Protein preparation

The protein of human centrin 2 (HsCen2) was cloned, expressed, and purified as previously reported.^46^ Using HsCen2 gene as template, HsCen2 was required by polymerase chain reaction (PCR) technique. The PCR product was connected to the pGEX-6p-1 vector enzymatically, and the cloning results were confirmed by DNA sequence detection. The recombinant plasmids were expressed, isolated, and purified by biotechnology according to a literature procedure.^40^ To remove the contaminating bound cations, the protein of HsCen2 was firstly pretreated with EDTA forming apoHsCen2 and then passed through a Sephadex G-25 column equilibrated in buffer 10 mM Hepes, at pH 7.4. Protein concentration of HsCen2 was measured using a molar extinction coefficient of 1490 M^−1^ cm^−1^ at 280 nm. The production of proteins was confirmed using SDS-PAGE.

HsCen2 was phosphorylated by PKA (0.5 μ μL^−1^) in 1.1 mM ATP, 5.0 mM MgCl_2_, and 10 mM Hepes (pH 7.4) at 30 °C for 5 h. The prepared sample was processed with a PD-10 column to remove excess ATP and Mg^2+^. The concentration of HsCen2p was measured using a molar extinction coefficient at 280 nm.

### 2.4 Aromatic residue-sensitized Tb^3+^ fluorescence

Aromatic residue-sensitized Tb^3+^ fluorescence experiments were carried out using HITACHI F-2700 spectrofluorometer in 10 mM Hepes at pH 7.4 and at room temperature. Both slit widths for excitation and emission were 10 nm. To avoid secondary Rayleigh scattering, the excitation wavelength was set at 295 nm, a 360 nm filter was used, and the Tb^3+^ emission fluorescence spectra were obtained in the range from 470 to 650 nm. The proteins (10 μM) were titrated with Tb^3+^ solution (1 mM), adding 5μ for each drop, and the corresponding emission spectra were recorded after incubation for 3 min.

The binding constant between the ligand (*L*) and HsCen2 can be calculated according to eqn (1)–(6), and the binding constant and the number of binding sites of protein − *L*_*n*_ can be obtained. Firstly, the free concentration is replaced by the total concentration of *L* in the calculation, which obviously will make calculations deviate from the actual result. Therefore, the free concentration of [*L*]_*f*_ is corrected by eqn (7). Then, fitting of log[*F*_0_ − *F*_i_]/[*F*_i_ − *F*_∞_] with log[*L*]_*f*_ was performed again by using the obtained [*L*]_*f*_, until the *K* value approached the approximate value for the *n*^th^ time and the (*n* + 1)^th^ time, which is the so-called iteration method. The more accurate binding constant and the number of binding sites of protein − *L*_*n*_ are calculated through iteration calculation.

1 protein + *nL* → protein − *L*_*n*_

2
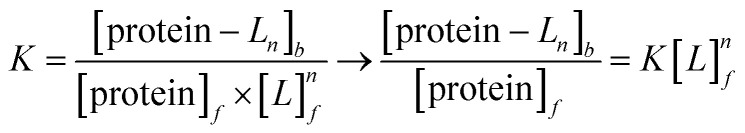

3 *F*_i_ − *F*_0_ ∝ [protein − *L*_*n*_]_*b*_

4 *F*_∞_ − *F*_i_ ∝ [protein]_*f*_

5
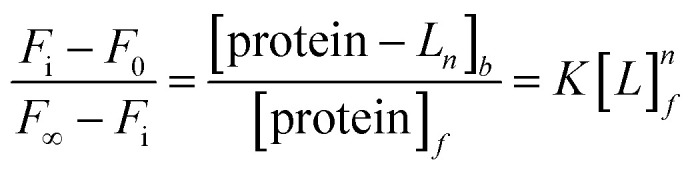
6
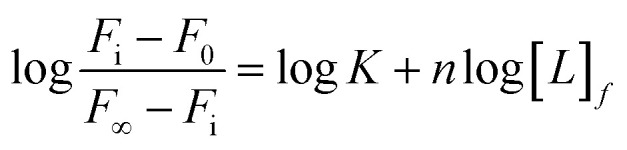


When *n* is 1,7
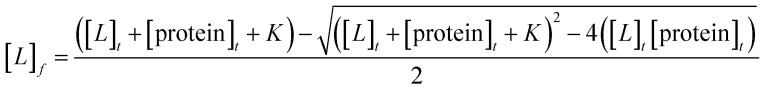


[*L*]_*f*_ is the free concentration of *L*, [protein]_*f*_ is the free concentration of protein, [protein − *L*_*n*_]_*b*_ is the complex final concentration of [protein − *L*_*n*_]. *F*_0_, *F* and *F*_∞_ represent the fluorescence intensity of proteins in the absence of *L*, in the presence of *L*, and the saturated intensity (at the saturation concentration), respectively. [*L*]_*t*_ is the total concentration of *L* and [protein]_*t*_ is the total concentration of protein.

When *n* is 2, it is assumed that the two binding sites are independent and identical in the protein. Firstly, [*L*]_*t*_ took the place of [*L*]_*f*_, fit of log[*F*_i_ − *F*_0_]/[*F*_∞_ − *F*_i_] against log[*L*]_*t*_ was performed using SigmaPlot 10.0 software to the two sites binding model, and the binding constants can be calculated according to eqn (6). Secondly, according to *K* and eqn (7), [*L*]_*f*_ was calculated. Then, fitting log[*F*_0_ − *F*_i_]/[*F*_i_ − *F*_∞_] against log[*L*]_*f*_ was performed again by using the obtained [*L*]_*f*_. The more accurate binding constant of protein with *L* is calculated through more iteration calculations.

### 2.5 Resonance light scattering measurements

The protein sample resonance light scattering (RLS) detection using synchronous fluorescence from 250–600 nm was analyzed by the HITACHI F-2700 spectrophotometer, and the excitation and the emission slits were set at 10 nm. The proteins (10 μM) were titrated with Tb^3+^ solution, and the corresponding RLS spectra were recorded after 3 min. The dilution effect of titration was deducted in the data processing. All fluorescence experiments were performed in 10 mM Hepes at pH 7.4 and room temperature.

### 2.6 Circular dichroism spectroscopy

Circular dichroism (CD) measurements of DNA interaction with proteins (HsCen2 or HsCen2p) in the absence or presence of Tb^3+^ were carried out with Applied Photophysics qCD (France) continuously purged by high N_2_ and equipped with a temperature-control system. CD spectra were recorded from 190 to 320 nm at room temperature using 1 mm quartz cells. Baseline correction was performed by subtracting the Hepes buffer.

### 2.7 Fluorescence spectroscopy

The interactions between the DNA with protein in the absence or presence of Tb^3+^ were measured using a HITACHI F-2700 spectrofluorometer. For the affinity of DNA towards protein (10 μM), the excitation wavelength was set at 280 nm. Titration was carried out with increasing concentrations of DNA (0–20 μM) to the protein of HsCen2 or HsCen2p, and the corresponding emission spectra were recorded. To detect the effect of Tb^3+^ on DNA binding to protein, protein (10 μM) was incubated with Tb^3+^ 1 : 2 for 10 min at 4 °C, and the Tb_2_-protein was titrated by gradually increasing the DNA concentration from 0 to 10 μM. The excitation wavelength was set to 295 nm, and the scanning range was 310–500 nm. Both the slit widths for excitation and emission were 10 nm. The reported constants are averages of three experiments.

Binding of the protein (HsCen2 or HsCen2p or Tb_2_-HsCen2 or Tb_2_-HsCen2p) with 2-*p*-toluidinylnaphthalene-6-sulfonate (TNS) was monitored by a fluorescence spectrometer. After incubation of the proteins with TNS for 10 min at 4 °C in the absence or presence of DNA, the mixed solutions were excited at 320 nm, and the scanning range was 330–650 nm.

### 2.8 Gel electrophoresis

0.005 μg μL^−1^ pBR322 DNA was mixed with protein or Tb_2_-protein, and the samples were incubated for a certain period of time. All samples were loaded in a 1% agarose gel, containing 4S Red Plus Nucleic Acid Stain. The electrophoresis was carried out at a constant pressure of 130 eV for about 30 min in 1×TAE (tris-acetic-EDTA). DNA reaction products were analyzed with the ultraviolet visible transmission reflectometer instrument (WFH-201BJ) at 300 nm.

### 2.9 Molecular docking simulation

All docking studies were carried out with ZDOCK 3.0.2 (https://zdock.umassmed.edu).^47^ The crystal structures of DNA duplex (PDB 1DJD) and HsCen2 (PDB 2GGM) were obtained from the PDB database. The modeled HsCen2p structure was established using Discovery Studio 2.5. For the prediction of possible complications, the server proceeds in three steps. Firstly, on the initial submission page, the user provides two input structures to be docked. The next step is the selection of residues for each submitted crystal structure, which is aided by JMol visualization of each molecule that highlights selected residues for the user. Finally, a link to the results is emailed to the user.

## 3 Results and discussion

### 3.1 Phosphorylation reduced the binding of protein to metal ions

#### 3.1.1 Förster resonance energy transfer between HsCen2 and Tb^3+^

Förster resonance energy transfer (FRET) is the physical process by which energy is transferred non-radiatively from an excited molecular chromophore (the donor, D) to another chromophore (the acceptor, A) by means of intermolecular long-range dipole–dipole coupling.^48,49^

Metal ion plays an important role in maintaining the advanced structure of the protein. Metal ions combine with appropriate proteins to form metalloproteins that drive different biological functions in the body.^42^

**Fig. 1:**
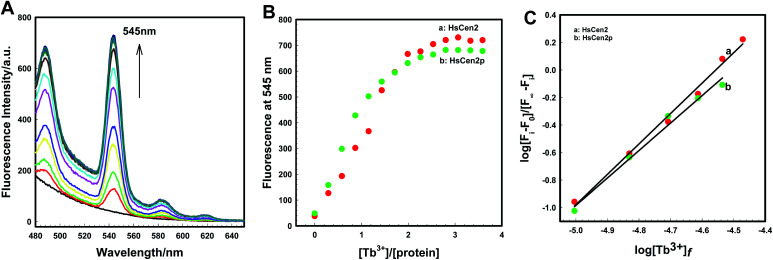
(A) Tb^3+^-sensitized fluorescence spectra of HsCen2 with increasing concentration of Tb^3+^ in 10 mM Hepes at pH 7.4. (B) Titration curves of fluorescence intensity for HsCen2 (a) and HsCen2p (b) at 545 nm against [Tb^3+^]/[protein] in 10 mM Hepes at pH 7.4. (C) The best fit of log[*F*_i_ − *F*_0_]/[*F*_∞_ − *F*_i_] at 545 nm against log[Tb^3+^]_*f*_ for HsCen2 (curve *a*) and HsCen2p (curve *b*), respectively.

In order to visually detect the interaction between Tb^3+^ and protein, FRET from the tyrosine residue on protein to bound Tb^3+^ was measured. When Tb^3+^ is added to HsCen2, the non-radiative energy transfer from tyrosine residue on HsCen2 to the bound Tb^3+^ resulted in the fluorescence sensitization of four weak characteristic peaks of Tb^3+^ at 490, 545, 585, and 620 nm (Fig. 1A). With increasing Tb^3+^ in HsCen2 solution, the fluorescence intensity of the four characteristic peaks of Tb^3+^ gradually increases. When the concentration of Tb^3+^ reaches saturation, the peak intensity does not change. Similarly, HsCen2p binding to Tb^3+^ displays the same phenomenon as HsCen2 (Fig. S1†). The titration curves are obtained by plotting the Tb^3+^-sensitized fluorescence intensity at 545 nm against [Tb^3+^]/[protein] (Fig. 1B). It can be seen that there are two sequential binding processes, each with a stoichiometric parameter *n* = 1. According to eqn (6), the binding constants can be calculated through three iterations, and the plots of log[*F*_i_ − *F*_0_]/[*F*_∞_ − *F*_i_] at 545 nm against the log[Tb^3+^]_*f*_ of HsCen2 (a) and HsCen2p (b) are shown in Fig. 1C. From Fig. 1C, the average binding constants of HsCen2 and HsCen2p can be calculated, *K*_HsCen2/Tb^3+^_, (1.10 ± 0.04) × 10^5^ M^−1^ and *K*_HsCen2p/Tb^3+^_, (3.10 ± 0.21) × 10^4^ M^−1^, respectively. The binding constants are listed in Table 1. The introduction of the phosphate group reduces the binding of Tb^3+^ to HsCen2.

**Table 1:** The binding constants of Tb^3+^ binding to HsCen2 or HsCen2p

Protein	*K* (error) (M^−1^)
HsCen2	(1.10 ± 0.04) × 10^5^^a^
	(1.60 ± 0.05) × 10^5^^b^
HsCen2p	(3.10 ± 0.21) × 10^4^^a^
	(1.40 ± 0.11) × 10^4^^b^

a Data from spectrofluorimetric titrations.

b Data from RLS titrations.

#### 3.1.2 HsCen2 RLS signal changes

**Fig. 2:**
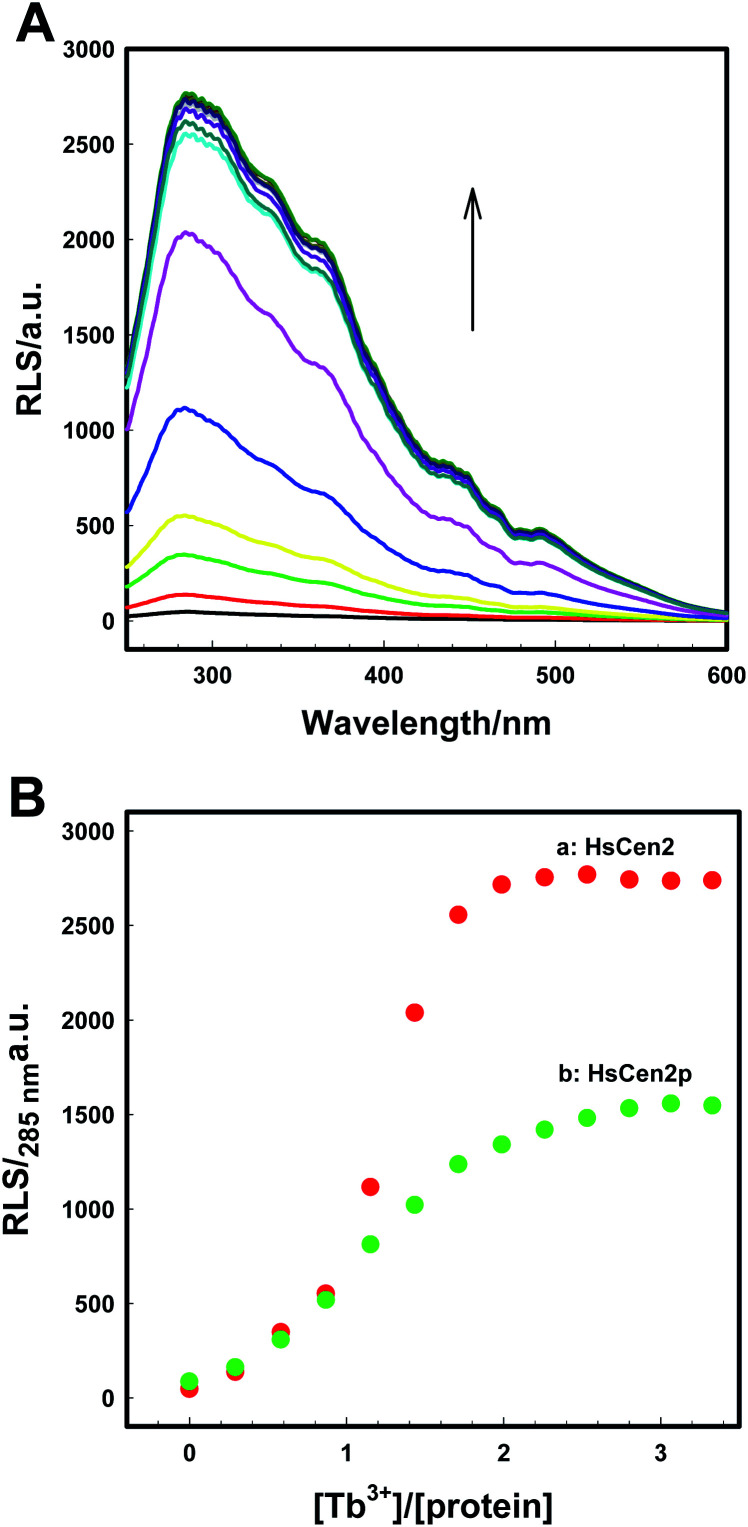
(A) Resonance light scattering (RLS) spectra of HsCen2 in the different concentrations of Tb^3+^. (B) Titrating curves of RLS intensity for HsCen2 (curve *a*) or HsCen2p (curve *b*) at 285 nm against [Tb^3+^]/[protein]. All the experiments in 10 mM Hepes (pH 7.4) at room temperature.

The size difference and charge-coupled transfer of the scatterers before and after the interaction due to the electrostatic or hydrophobic interactions will result in the enhancement of the RLS signal.^50^ In addition, the change in ionic strength of the medium would change the charges of both interacting components and the conformation of biological molecules, which will also cause the change in the RLS signal.^50^ The RLS spectra of the interaction between Tb^3+^ and HsCen2 are shown in Fig. 2A. RLS signal continues to increase with the increase in Tb^3+^ concentration. The combination of HsCen2p and Tb^3+^ also shows the same phenomenon as HsCen2 (Fig. S2A†). Fig. 2B shows the titration curves of RLS intensity of proteins at 285 nm against [Tb^3+^]/[protein], in which curve a is the titration curve of HsCen2 and curve *b* is the titration curve of HsCen2p. From Fig. 2B curve *a*, the RLS signal increases gradually from 0 to 1 of [Tb^3+^]/[HsCen2]. With the further addition of Tb^3+^, the RLS signal increases continuously. In the range of 1 to 2 for [Tb^3+^]/[HsCen2], the RLS signal increased remarkably, and beyond [Tb^3+^]/[HsCen2] above 2, the signal intensity of RLS reached the maximum and did not change with increasing Tb^3+^ concentration. The binding ratio of Tb^3+^ to HsCen2 is 2 : 1. After phosphorylation modification, the RLS intensity of HsCen2p was significantly reduced compared to HsCen2 (curve *b* in Fig. 2B). Fig. S2B† are the plots of log[RLS_i_ − RLS_0_]/[RLS_∞_ − RLS_i_] at 285 nm against the log[Tb^3+^]_*f*_ of HsCen2 (a) and HsCen2p (b), with the data derived from Fig. 2B. The evaluated average binding constants are calculated to be *K*_HsCen2/Tb^3+^_, (1.60 ± 0.05) × 10^5^ and *K*_HsCen2p/Tb^3+^_, (1.40 ± 0.11) × 10^4^ M^−1^ (Fig. S2B†). Detailed binding constants are listed in Table 1. They are consistent with the results of Tb^3+^ sensitization fluorescence.

HsCen2 can bind to two Tb^3+^, and the binding of Tb^3+^ can cause the RLS signal enhancement. After phosphorylation modification, the binding of Tb^3+^ to HsCen2p is weakened, and the binding constant is reduced by at least one order of magnitude. The RLS intensity of HsCen2 induced by Tb^3+^ is reduced by phosphorylation by about 46%, *i.e.*, phosphorylation inhibits the RLS intensity induced by Tb^3+^.

#### 3.1.3 Tb^3+^-induced secondary changes of HsCen2

**Fig. 3:**
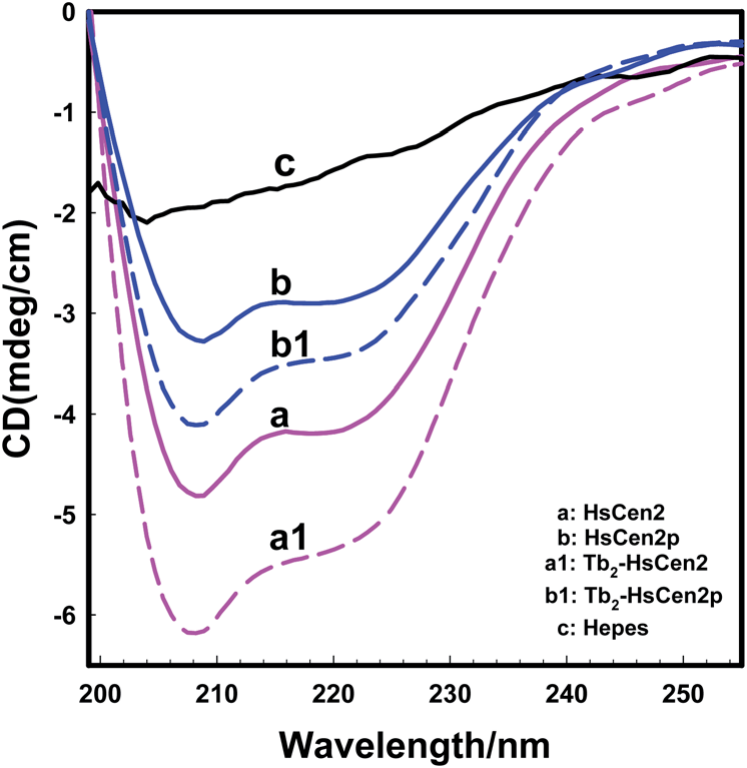
Far-UV CD spectra of HsCen2 (curve *a*) or Tb_2_-HsCen2 (Tb^3+^-saturated HsCen2, curve a1) or HsCen2p (curve *b*) or Tb_2_-HsCen2p (curve b1) using 1 mm path length quartz cells in 10 mM Hepes, pH 7.4 at 25 °C, curve *c* is a blank control.

The binding of metal ions can induce the conformation change of protein. As shown in Fig. 3, the far-UV CD spectrum of HsCen2 (curve *a*) showed two negative peaks at 208 and 222 nm, which is typical for a well-folded protein with an α-helical secondary structure.^11^ The change in molar ellipticity (monitored at 208 nm) occurred upon the addition of 2 equivalent Tb^3+^ (curve a1), with the change in molar ellipticity of HsCen2 increasing by 20.4%. Curve *b* is the CD spectrum of HsCen2p. The binding of HsCen2p to Tb^3+^ also leads to an increase in the α-helical content of the protein after adding 2 equivalent Tb^3+^ (curve b1) to HsCen2p, with the change in molar ellipticity of HsCen2p increasing by 9.7%. Compared with the changes in α-helix content induced by Tb^3+^ binding before and after phosphorylation of HsCen2, the results showed that the introduction of the phosphate group weakened the conformational changes of HsCen2 induced by Tb^3+^.

The molecular interactions between proteins are mediated probably by electrostatic and hydrophobic forces.^51–53^ Due to the phosphorylation modification of the protein, the negative charge of the protein increases, and the exposure of the hydrophobic cavity decreases,^41^ which changes the electrostatic and hydrophobic interaction between the proteins. So, phosphorylation not only reduces the binding of Tb^3+^ to HsCen2 but also changes the conformation of HsCen2 induced by Tb^3+^.

### 3.2 The binding of protein to DNA and its endonuclease-like activity

#### 3.2.1 The characterization of protein interacting with DNA

**Fig. 4:**
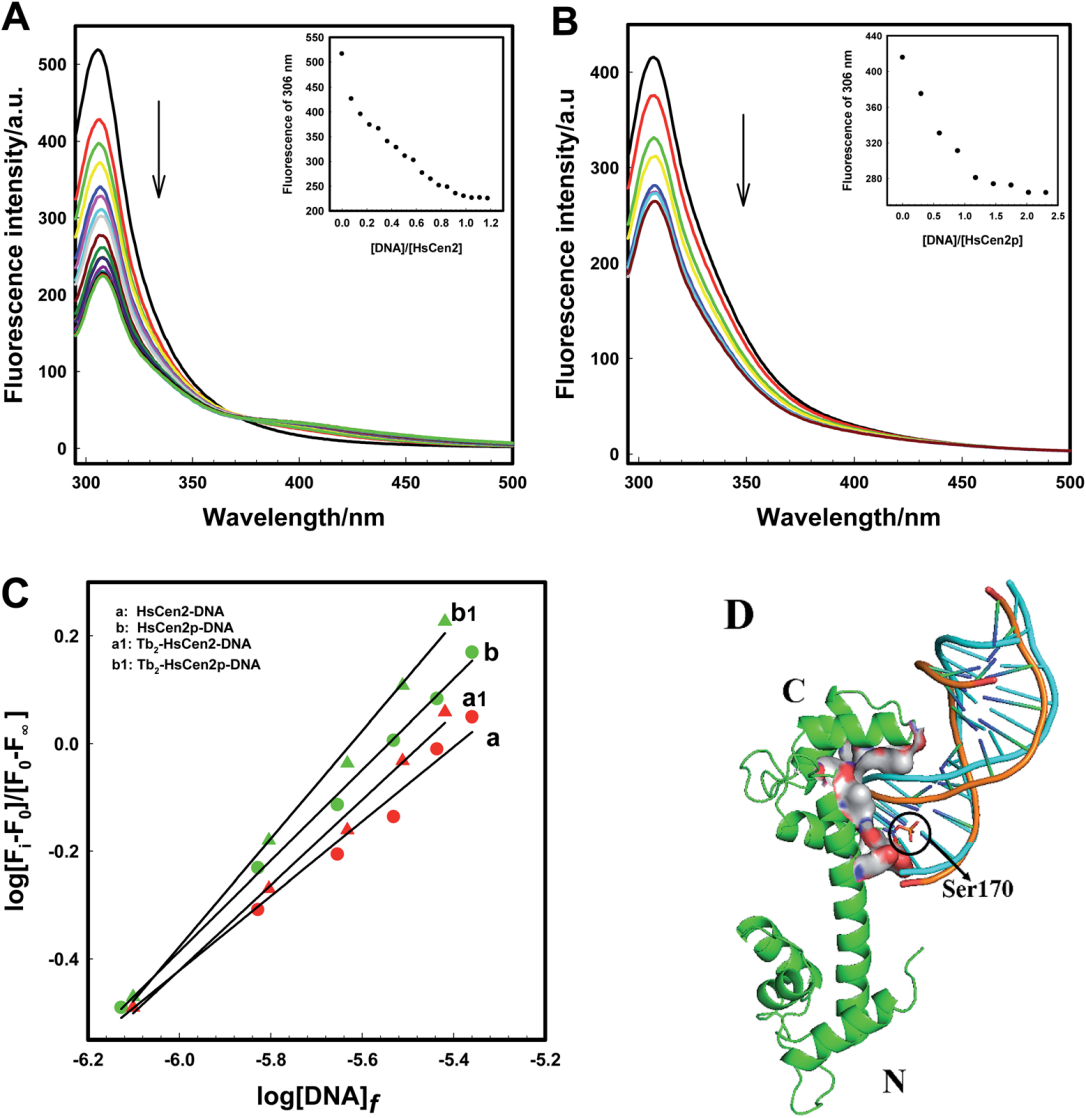
Fluorescence spectra of HsCen2 (A) and HsCen2p (B) with the increase in DNA concentration in 10 mM Hepes at pH 7.4. Inset, the titrating curves of fluorescence intensity for HsCen2(A) or HsCen2p (B) at 306 nm against [DNA]/[protein]. (C) The best fits of log[*F*_i_ − *F*_0_]/[*F*_∞_ − *F*_i_] at 306 nm against log[DNA]_*f*_ for HsCen2 (a) or Tb_2_-HsCen2 (a1) or HsCen2p (b) or Tb_2_-HsCen2p (b1), respectively. (D) Schematic representation of HsCen2 (PDB 2GGM) interaction with DNA (PDB 1DJD, cartoon: yellowish-brown) and modeled HsCen2p interaction with DNA (cartoon: blue) by ZDOCK 3.0.2. Ser is shown as stick models (Ser170 is in yellowish-brown sticks).

The analysis of the binding property of HsCen2 to DNA is useful for understanding the function of centrin. To verify the effect of phosphorylation on the binding property of HsCen2 to DNA in the absence or presence of Tb^3+^, the fluorescence spectra of the tyrosine luminescence of HsCen2 at 306 nm were also used. As revealed in Fig. 4A, with the additional concentration of DNA, the fluorescence peak of HsCen2 at 306 nm is quenched gradually. The inset of Fig. 4A shows the titration curve obtained from the plot of fluorescence intensity of HsCen2 at 306 nm against [DNA]/[HsCen2]. With the addition of DNA and when [DNA]/[HsCen2] is 1, the titration curve gradually flattens out. The fluorescence intensity of HsCen2 is quenched to a minimum and no longer changes. It means that HsCen2 and DNA form a complex, and one molecule of protein can bind to one DNA. The fluorescence spectra of the interaction between HsCen2p and DNA are shown in Fig. 4B. It can be seen that with the increase in DNA concentration, the fluorescence intensity of HsCen2p at 306 nm is continuously quenched, and an inflection point appears near the ratio of [DNA]/[HsCen2p] = 1 (inset of Fig. 4B). In the presence of metal ions (Tb^3+^), the same trend of interaction between proteins and DNA is shown (the fluorescence spectra are shown in Fig. S3†). The plots of log[*F*_i_ − *F*_0_]/[*F*_∞_ − *F*_i_] at 306 nm against log[DNA]_*f*_ for HsCen2 (curve *a*) or Tb_2_-HsCen2 (curve a1) or HsCen2p (curve *b*) or Tb_2_-HsCen2p (curve b1) are shown in Fig. 4C. According to the method given in the experimental part, after more iterations, the binding constant of HsCen2 or HsCen2p or Tb_2_-HsCen2 or Tb_2_-HsCen2p with DNA can be obtained, *K*_HsCen2/DNA_, (6.30 ± 0.01) × 10^3^ or *K*_HsCen2p/DNA_, (5.70 ± 0.03) × 10^4^ or *K*_Tb_2_-HsCen2/DNA_, (2.63 ± 0.04) × 10^4^ or *K*_Tb_2_-HsCen2p/DNA_, (6.30 ± 0.05) × 10^5^ M^−1^, respectively. The binding constants are listed in Table 2. The results showed that phosphorylation promotes the binding of DNA to HsCen2, and the presence of metal ions is further beneficial to the binding of DNA to protein.

**Table 2:** The binding constants of DNA interaction with different proteins (data from spectrofluorimetric titrations)

Protein	*n*	*K* (error) (M^−1^)
HsCen2	0.85	(6.30 ± 0.01) × 10^3^
HsCen2p	0.70	(5.70 ± 0.03) × 10^4^
Tb_2_-HsCen2	0.73	(2.63 ± 0.04) × 10^4^
Tb_2_-HsCen2p	0.98	(6.30 ± 0.05) × 10^5^

The binding mode of protein and DNA is mainly through the hydrogen bond or its complementary spatial structure with DNA structure or DNA special base sequence combination.^54^Fig. 4D is the overall superposition model structure of the interaction between HsCen2 and HsCen2p with DNA using ZDOCK 3.0.2. Structurally, the interaction between HsCen2 and DNA (cartoon: yellowish-brown) is such that the phosphate skeleton of DNA is stuck in the cavity formed between the EF3 and EF4 domains of HsCen2, and Ser170 in the C-terminal of HsCen2 is close to the base pair of the DNA. Although the way of interaction between HsCen2p and DNA (cartoon: blue) does not change significantly, there is a little difference in the direction and the position of DNA, which is shifted up. Since the phosphorylation modification introduces two units of negative charge at the Ser170 of HsCen2, the electrostatic force between the positive electricity of base pairs and Ser170 enhances and makes the binding between DNA and HsCen2p stronger than HsCen2. In addition, the binding of metal ions changes the conformation of protein from a “closed” to an “open” state, and the exposure of hydrophobic cavity increases,^11^ which is more conducive to the binding of DNA and protein.

#### 3.2.2 Conformational changes of DNA and protein

**Fig. 5:**
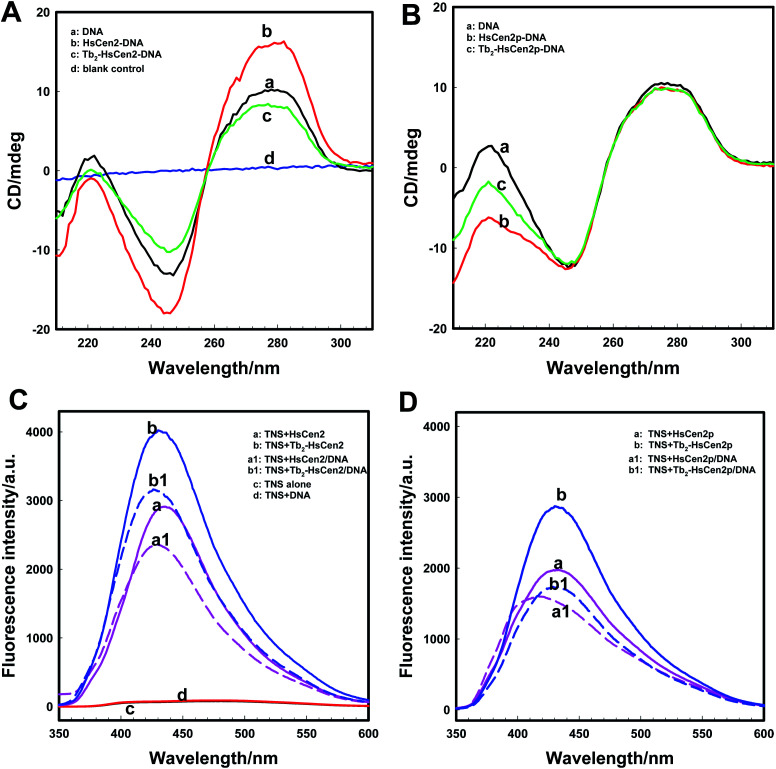
HsCen2 binding DNA. Far-UV CD spectra of DNA bound to HsCen2 (A, 5 μM) or HsCen2p (B, 5 μM) in the absence or presence of Tb^3+^ (10 μM) in 10 mM Hepes, pH 7.4, the concentration of DNA was 5 μM. The Tb^3+^ and protein mixed solution was equilibrated for 5 min before the addition of DNA. (A) Curve *a*: DNA alone in buffer, curve *b*: DNA + HsCen2, curve *c*: DNA + Tb_2_-HsCen2, curve *d*: blank control. (B) Curve *a*: DNA alone in buffer, curve *b*: DNA + HsCen2p, curve *c*: DNA + Tb_2_-HsCen2p. (C) Fluorescence spectra of TNS interacting with different proteins of HsCen2 (a, 10 μM) or HsCen2/DNA complex (a1, 10 μM) or Tb_2_-HsCen2 (b, 10 μM) or Tb_2_-HsCen2/DNA complex (b1, 10 μM). TNS alone (c, 100 μM) and TNS + DNA (d, 10 μM) excited at 320 nm in 10 mM Hepes at pH 7.4. (D) Fluorescence spectra of TNS in the presence of HsCen2p (a, 10 μM) or HsCen2p/DNA complex (a1, 10 μM) or Tb_2_-HsCen2p (b, 10 μM) or Tb_2_-HsCen2p/DNA complex (b1, 10 μM) excited at 320 nm in 10 mM Hepes at pH 7.4.

The structural changes of proteins induced environmentally were determined using CD spectroscopy, which is based on the different forms of primary and secondary structural elements found in proteins.^35^ The binding of DNA with HsCen2 was monitored using CD spectra. It can be seen from Fig. 5A (curve *a*) that DNA has a positive peak at 277 nm due to base stacking, and the right-handed helix of DNA has a negative peak at 245 nm, which is the characteristic peak of a typical B-type DNA structure.^36^ The drastic change can be observed after adding HsCen2 to DNA (curve *b*). The negative peak at 245 nm and the positive peak at 277 nm enhanced significantly due to the addition of HsCen2, indicating that the double helix structure of DNA changes and a new complex of HsCen2/DNA may form. Similar to the binding of HsCen2 to DNA, after mixing Tb_2_-HsCen2 with DNA (curve *c*), the DNA negative peak at 245 nm and the positive peak at 277 nm were weakened, which might be because of the presence of Tb^3+^ damaging the structure of DNA. The results also showed that Tb_2_-HsCen2 also interacted with DNA.

As for as HsCen2p (Fig. 5B), HsCen2p displays different effects on DNA double helix to HsCen2. Comparing curve *a* and curve *b* in Fig. 5B, when HsCen2p is mixed with DNA, no obvious conformational changes from DNA at 277 nm have been obtained. However, the negative peak at 245 nm is broadened and blue-shifted to 243 nm, which is a characteristic of the destruction of the double helix structure of DNA. It shows that HsCen2p forms a complex with DNA to change the double helix structure of DNA. After mixing Tb_2_-HsCen2p with DNA (curve *c*), the negative peak at 245 nm also becomes wider and changes the double helix structure of DNA. It indicates that Tb_2_-HsCen2 also interacts with DNA.

Conformational changes of protein caused by the addition of DNA were detected by TNS fluorescence emission. The exposure of hydrophobic surfaces on HsCen2 or HsCen2p or Tb_2_-HsCen2 or Tb_2_-HsCen2p after binding with DNA was investigated. Fig. 5C is the fluorescence spectra of TNS interaction with HsCen2 or Tb_2_-HsCen2 in the absence or presence of DNA. As shown in Fig. 5C, the TNS alone fluorescence is weak at 500 nm in Hepes (curve *c*). After binding with HsCen2 (curve *a*), the fluorescence emission peak of TNS increased significantly with a blue-shift from 500 nm to about 435 nm. It can be inferred that the micro-environments of TNS were changed from polar to non-polar.^27^ Compared with TNS binding to HsCen2, after binding TNS to HsCen2/DNA complex (curve a1), the fluorescence emission decreased significantly with a blue shift (6 nm) from 435 to 429 nm. This may be due to the fact that DNA binds to the protein and occupies the hydrophobic cavity of the protein, reducing the exposure of the hydrophobic cavity of the protein. After Tb^3+^ binds to the protein, the conformation of the protein changes from a “closed” to an “open” state, and the exposure of the hydrophobic cavity increases.^11^ Curve *b* is the spectra of the interaction between TNS and Tb_2_-HsCen2. Compared with curve a, the fluorescence of TNS is slightly increased, indicating that the exposure of the hydrophobic cavity of HsCen2 is increased, which is consistent with the previous conclusion. After binding TNS to the Tb_2_-HsCen2/DNA complex (curve b1) the fluorescence emission decreased significantly with a blue shift (7 nm) from 435 to 428 nm. The results show that the presence of metal ions makes HsCen2 in a more hydrophobic environment, which is conducive to the binding of HsCen2 to DNA.

Fluorescence spectra of TNS interaction with HsCen2p or Tb_2_-HsCen2p in the presence or absence of DNA are shown in Fig. 5D. From Fig. 5D, it can also be concluded that the binding of DNA reduces the exposure of the hydrophobic cavity of the protein, and the metal ions have a certain promotion effect on the binding of the protein and DNA. Comparing curve a in Fig. 5C and D, the fact that fluorescence emission of TNS in the presence of HsCen2p is weaker than that in the presence of HsCen2 shows that the introduction of the phosphate group on HsCen2 leads to a decrease of hydrophobic areas on protein.^27^ The fluorescence emission of TNS combined with HsCen2p/DNA (curve a1) shows a blue shift from 431 to 417 nm (16 nm). Comparing the conformational changes of HsCen2 binding to DNA (blue shift 6 nm) and HsCen2p binding to DNA (blue shift 16 nm) (curve a1 in Fig. 5C and D), it can be concluded that the introduction of phosphate groups makes the environment of the protein more hydrophobic after the DNA binds to protein.

#### 3.2.3 Phosphorylation promoting the endonuclease-like activity of HsCen2

**Fig. 6:**
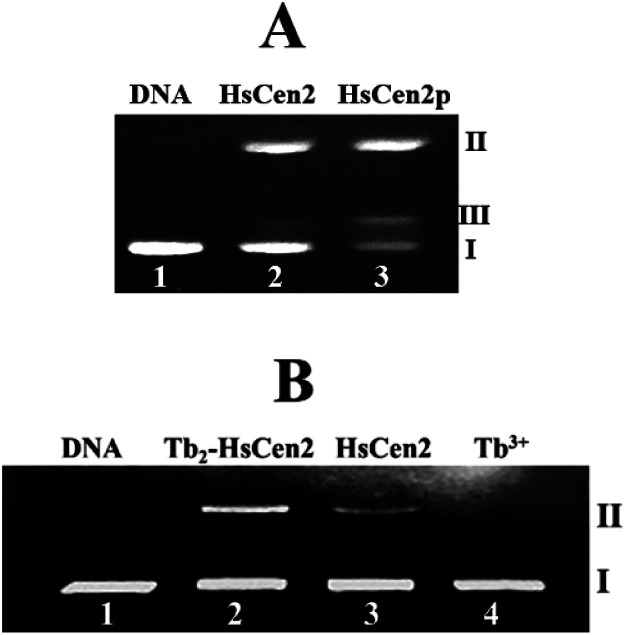
(A) The agarose gel electrophoresis of pBR322 DNA (0.005 μg μL^−1^) after incubation with HsCen2 (35 μM) or HsCen2p (35 μM) for 2 h at 4 °C in 10 mM Hepes buffer (pH 7.4), lane 1: control untreated supercoiled DNA, lane 2: DNA + HsCen2, lane 3: DNA + HsCen2p. (B) Agarose gel electrophoresis of pBR322 DNA (0.005 μg μL^−1^) after incubation with different species for 2 h in 10 mM Hepes buffer (pH 7.4) at 4 °C. Lane 1: control untreated supercoiled DNA, lane 2: DNA + Tb_2_-HsCen2, lane 3: DNA + HsCen2, lane 4: DNA + Tb^3+^, [Tb^3+^] = 60 μM, [protein] = 30 μM.

As previously reported, EoCen has endonuclease-like activity, and the cleavage mechanism of EoCen to pBR322 DNA might be the hydrolysis mechanism.^55^ HsCen2 has 62% sequence homology with EoCen, and HsCen2 is involved in the NER process.^56,57^ Whether HsCen2 has similar endonuclease-like activity to EoCen is worthy of investigation. Therefore, the endonuclease-like activity and the regulation of phosphorylation on the endonuclease-like activity of HsCen2 were explored. HsCen2/HsCen2p (35 μM) and pBR322 DNA (0.005 μg μL^−1^) were incubated at 4 °C for 2 h. Agarose gel electrophoresis results are shown in Fig. 6A. pBR322 DNA is a supercoiled nucleic acid macromolecule (*i.e.*, Form I DNA). From Fig. 6A, it can be seen that Form I DNA (supercoiled DNA) (lane 1) is cleaved by HsCen2 into Form II DNA (a circular DNA strand with a gap) (lane 2), while HsCen2p can cleave Form I DNA into Form II and Form III DNA (linear DNA) (lane 3). Obviously, the endonuclease-like activity of HsCen2p is much higher than that of HsCen2 under the same conditions. The results of agarose gel electrophoresis of the hydrolysis of pBR322 DNA by HsCen2 in the absence or presence of Tb^3+^ are shown in Fig. 6B. The pBR322 DNA (0.005 μg μL^−1^) was incubated with Tb_2_-HsCen2 (lane 2) and HsCen2 (lane 3) (30 μM) for 2 h at 4 °C. As can be seen from Fig. 6B, Form I DNA (lane 1) is cleaved by HsCen2 or Tb_2_-HsCen2 into Form II DNA. However, the content of Form II DNA increased significantly in the presence of Tb^3+^. In contrast, under the same conditions and in the presence of metal ions (lane 4), pBR322 DNA did not produce any split products. The results showed that the presence of Tb^3+^ increased the endonuclease activity of HsCen2.

**Fig. 7:**
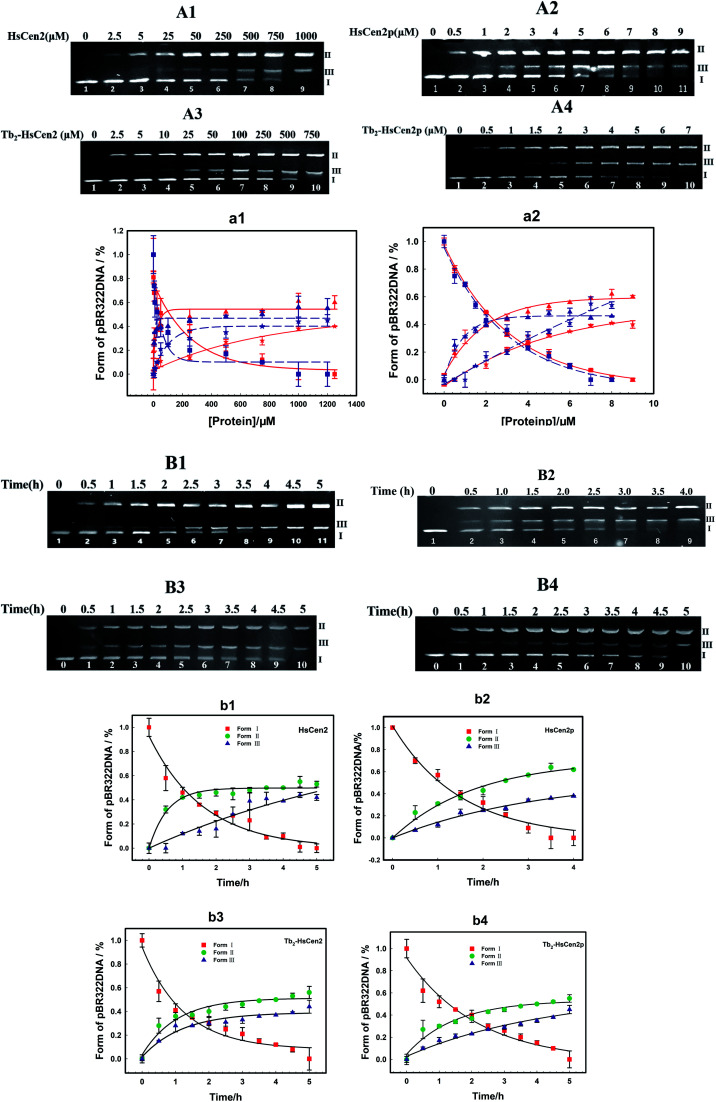
(A) Agarose gel electrophoresis of pBR322 DNA (0.005 μg μL^−1^) after incubation with HsCen2 (A1) or HsCen2p (A2) or Tb_2_-HsCen2 (A3) or Tb_2_-HsCen2p (A4) for 5 h at 4 °C. (a1) The plots of DNA content of Form I (red square is HsCen2, and the blue square is Tb_2_-HsCen2), Form II (red triangle represents HsCen2 and the blue triangle is Tb_2_-HsCen2) and Form III (red pentagram represents HsCen2 and the blue pentagram is Tb_2_-HsCen2) against concentrations of HsCen2 and Tb_2_-HsCen2, data derived from (A1 and A3), red, blue lines are the fitting results according to single-exponential fitting procedures. (a2) The plots of DNA content of Form I (red square is HsCen2p, and the blue square is Tb_2_-HsCen2p), Form II (red triangle represents HsCen2p and the blue triangle is Tb_2_-HsCen2p) and Form III (red pentagram represents HsCen2p and the blue pentagram is Tb_2_-HsCen2p) against the concentration of HsCen2p and Tb_2_-HsCen2p, with data from (A2 and A4), red, blue lines are the fitting results according to single-exponential fitting procedures. (B) Agarose gel electrophoresis of pBR322 DNA (0.005 μg μL^−1^) after incubation with HsCen2 (B1, 1 mM) or HsCen2p (B2, 9 μM) or Tb_2_-HsCen2 (B3, 0.5 mM) or Tb_2_-HsCen2p (B4, 4 μM) for different times, respectively. The plots of DNA content of Form I, Form II, and Form III against time for HsCen2 (b1) or HsCen2p (b2) or Tb_2_-HsCen2 (b3) or Tb_2_-HsCen2p (b4), data derived from (B1–B4).

Next, the optimal conditions for different proteins of HsCen2 or HsCen2p or Tb_2_-HsCen2 or Tb_2_-HsCen2p to cleave the supercoiled DNA were explored. The agarose gel electrophoresis results of DNA cleavage after incubation with different concentrations of proteins of HsCen2 or HsCen2p or Tb_2_-HsCen2 or Tb_2_-HsCen2p and DNA for 5 h are shown in Fig. 7A1–A4. Different concentrations of HsCen2 and DNA were incubated at 4 °C for 5 h, and the agarose gel electrophoresis results are shown in Fig. 7A1. When HsCen2 is not added, the supercoiled DNA is a complete band (lane 1); when 250 μM HsCen2 is added, supercoiled DNA is cleaved into notched and linear DNA (lane 6); when 1000 μM of HsCen2 is added, the supercoiled DNA disappears and is completely cleaved into notched and linear DNA (lane 9). Similarly, we analyzed the concentration gradient of HsCen2p and DNA incubated at 4 °C for 5 h. From Fig. 7A2, we found that while 9 μM of HsCen2p is added, the supercoiled DNA disappears and is completely cleaved into notched and linear DNA. By comparing Fig. 7A1 and A2, after protein phosphorylation, the protein concentration of 9 μM can completely cleave DNA. It can be concluded that the introduction of phosphate groups greatly enhanced the endonuclease-like activity of HsCen2. The agarose gel electrophoresis results of pBR322 DNA cleavage by Tb_2_-HsCen2 (0–750 μM) and Tb_2_-HsCen2p (0–7 μM) at different concentrations are shown in Fig. 7A3 and A4. The results showed that the presence of Tb^3+^ is conducive to the cleavage of DNA by proteins.

The plots of contents of Form I, Form II, and Form III DNA against the concentration of HsCen2 or Tb_2_-HsCen2 are shown in Fig. 7a1, and against the concentration of HsCen2p or Tb_2_-HsCen2p are shown in Fig. 7a2, respectively. It is assumed that Form I supercoiled DNA is cleaved by 50%, and the required protein concentration is defined as the IC_50_ value. The IC_50_ of HsCen2 is 113.86 μM, and the IC_50_ of Tb_2_-HsCen2 is 30.62 μM. The IC_50_ values are listed in Table 3. After phosphorylation, the IC_50_ of HsCen2p is 1.93 μM, and the IC_50_ of Tb_2_-HsCen2 is 1.79 μM. Since the concentration of phosphorylated protein required for complete hydrolysis of DNA is very small, IC_50_ has little effect in the absence or presence of Tb^3+^. The concentration of HsCen2 is 59 times higher than that of HsCen2p. Phosphorylation significantly increases the endonuclease-like activity of HsCen2 and further supports the strong binding ability of HsCen2p to DNA (Table 2).

**Table 3:** Protein concentration when different proteins catalyze DNA hydrolysis by 50% (IC_50_ value)

Protein	IC_50_ (μM)
HsCen2	113.86
HsCen2p	1.93
Tb_2_-HsCen2	30.62
Tb_2_-HsCen2p	1.79

To further evaluate the ability of HsCen2 or HsCen2p or Tb_2_-HsCen2 or Tb_2_-HsCen2p to cleave pBR322 DNA, DNA cleavage by proteins over time was explored (Fig. 7B1–B4). Fig. 7B1 is the agarose gel electrophoresis results of HsCen2 (1000 μM) and DNA (0.005 μg μL^−1^) incubation at different times. With the increase in incubation time, Form I of supercoiled DNA is cleaved to Form II DNA; then, the Form II DNA is again cleaved into Form III, linear DNA. When incubation time reaches 4.5 h, the Form I of supercoiled DNA is completely cleaved by HsCen2 and achieves the best endonuclease-like activity. The time gradient agarose gel electrophoresis results of HsCen2p (9 μM) cleavage pBR322 DNA (0.005 μg μL^−1^) are shown in Fig. 7B2. It can be seen that only in 4 h, Form I supercoiled DNA can be completely cleaved by HsCen2p. The gel electrophoresis results of the time gradient for Tb_2_-HsCen2 or Tb_2_-HsCen2p cleavage of DNA are shown in Fig. 7B3 and B4. The results showed that only in 5 h supercoiled DNA can be completely cleaved by HsCen2p (500 μM) or Tb_2_-HsCen2p (4 μM), respectively.

The plots of DNA content of Form I, Form II, and Form III against time for HsCen2 (b1) or HsCen2p (b2) or Tb_2_-HsCen2 (b3) or Tb_2_-HsCen2p (b4) are shown in Fig. 7b1–b4, with the data from (B1–B4). Taking the Form I supercoiled DNA cleavage as a marker, the curves, of which the contents of Form I supercoiled DNA plots against the time, are fitted with the aid of a single exponential fitting program. For the convenience of comparison, the rate constant is converted into a molar rate constant. The constant for HsCen2p (9.00 ± 2.40) × 10^4^ M^−1^ h^−1^) is 145 times that of HsCen2 (0.62 ± 0.06) × 10^3^ M^−1^ h^−1^), and the constant for Tb_2_-HsCen2 (1.73 ± 0.06) × 10^3^ M^−1^ h^−1^) is 2.8 times that of HsCen2 (0.62 ± 0.06) × 10^3^ M^−1^ h^−1^) (Table 4). It can be concluded that both phosphorylation and Tb^3+^ binding can increase the endonuclease-like activity of HsCen2.

**Table 4:** Rate constants of DNA hydrolysis by different proteins

Protein	*k*_cat_ (error) 10^3^ (h^−1^ M^−1^)
HsCen2 (1 mM)	0.62 ± 0.06
HsCen2p (9 μM)	74.48 ± 0.05
Tb_2_-HsCen2 (0.5 mM)	1.73 ± 0.06
Tb_2_-HsCen2p (4 μM)	234.00 ± 0.09

**Fig. 8:**
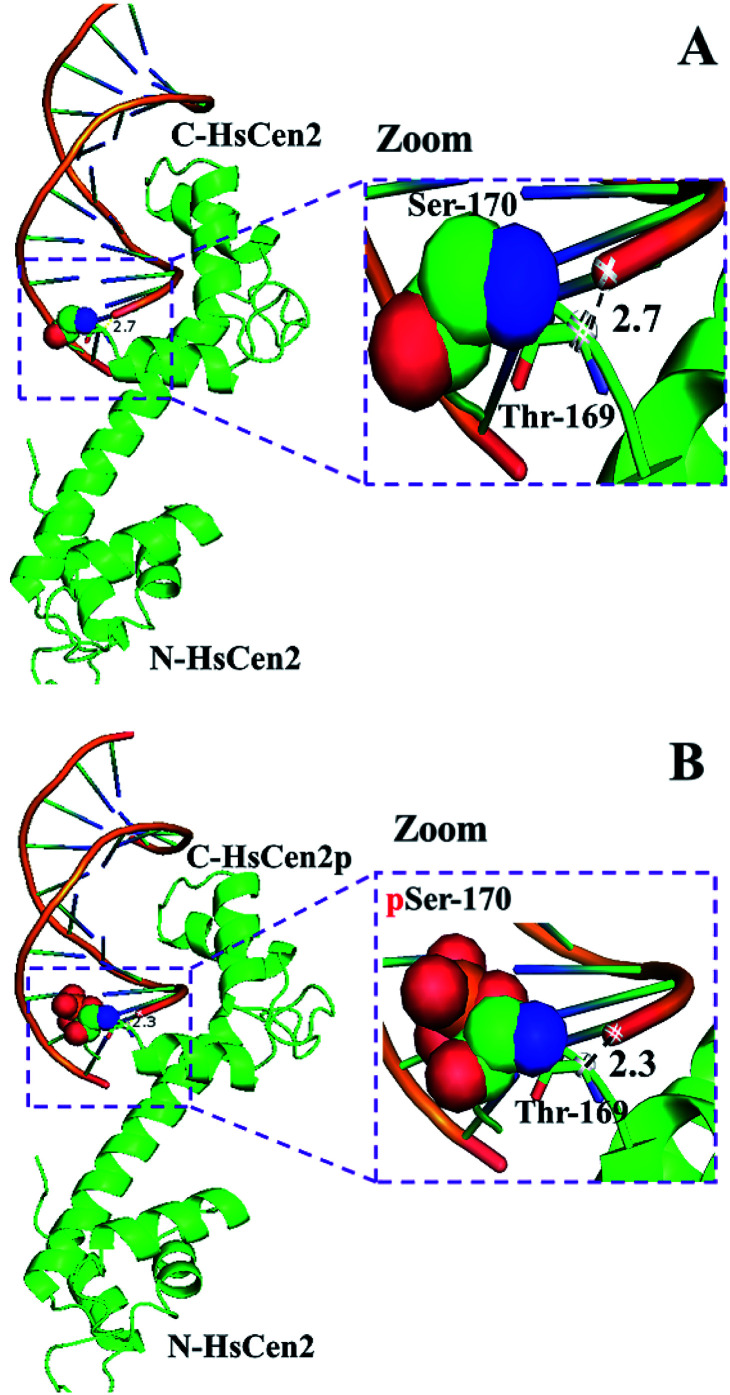
The model structure of HsCen2 or HsCen2p interaction with DNA (PDB 1DJD). The distance between Thr169 of HsCen2 (A) or HsCen2p (B) and the DNA phosphate center is shown in the dash line, respectively.

DNA binding is a critical step for subsequent cleavage. The hydrolysis of DNA by centrin is related to Ser and/or Thr in the protein.^40^ The hydroxyl groups of Ser and/or Thr attack the phosphorus center in a nucleophilic way, resulting in the formation of a pentacoordinate phosphorus transition state and efficient DNA cleavage taking place.^40^ When the HsCen2 is combined with DNA, the hydroxyl group of Thr169 is close to the phosphorus center of the phosphate skeleton in DNA. The distance between the carbon (–CH–OH) of Thr169 and the phosphorus of the phosphate skeleton in DNA is 2.7 Å (Fig. 8A). If Thr169 is the active center of the endonuclease-like activity of HsCen2, the hydroxyl group of Thr169 attacks the phosphorus center in a nucleophilic way and causes the cleavage of the DNA. After phosphorylation, the distance between the carbon (–CH–OH) of Thr169 and the phosphorus of the phosphate skeleton in DNA becomes 2.3 Å (Fig. 8B); the hydroxyl group of Thr169 easily attacks the phosphorus center in a nucleophilic way and results in the hydrolysis of DNA efficiently. Phosphorylation enhances the endonuclease-like activity of HsCen2. After the protein binds to metal ions, the conformational changes that occur are more conducive to the protein cleaving the DNA. In addition, the binding of Tb^3+^ to the protein may facilitate the ionization of adjacent residues of the protein, which will also affect the cleavage efficiency of the protein.^58,59^

## 4 Conclusions

In summary, phosphorylation regulates the properties of HsCen2, including the binding of metal ions and participation in the process of NER. The average binding constants of Tb^3+^ to HsCen2 or HsCen2p are 1.10 × 10^5^ M^−1^ and 3.10 × 10^4^ M^−1^, respectively. The introduction of the phosphate group weakened the binding of Tb^3+^ to HsCen2 and reduced the binding capacity by 1 order of magnitude. DNA can form a 1 : 1 complex with HsCen2 or HsCen2p with the binding constant of 6.30 × 10^3^ M^−1^ and 5.70 × 10^4^ M^−1^, respectively. Phosphorylation enhances the binding of DNA to HsCen2. HsCen2 shows a certain endonuclease-like activity that could hydrolyze DNA. The rate constants of HsCen2 and HsCen2p catalyzing supercoiled DNA are 0.62 × 10^3^ h^−1^ M^−1^ and 90.0 × 10^3^ h^−1^ M^−1^, respectively. Phosphorylation improves the endonuclease-like activity of HsCen2. Additionally, in the presence of Tb^3+^, it will enhance the binding of DNA to protein and improve the endonuclease-like activity of the protein.

## Conflicts of interest

There are no conflicts to declare.

## Supplementary Material

RA-012-D2RA03402F-s001

## References

[cit1] Huang B., Mengersen A., Lee V. D. (1988). J. Cell Biol..

[cit2] Sanders M. A., Salisbury J. L. (1994). J. Cell Biol..

[cit3] Zhang W. L., Shi E. X., Zhao Y. Q., Yang B. S. (2018). J. Inorg. Biochem..

[cit4] Kilmartin J. V. (2003). J. Cell Biol..

[cit5] Zhao Y. Q., Guo X. J., Yang B. S. (2017). RSC Adv..

[cit6] Lewit-Bentley A., Réty S. (2000). Curr. Opin. Struct. Biol..

[cit7] Durussel I., Blouquit Y., Middendorp S., Cracscu C. T., Cox J. A. (2000). FEBS Lett..

[cit8] Grant B. M. M., Enomoto M., Ikura M., Marshall C. B. (2020). Int. J. Mol. Sci..

[cit9] Matei E., Miron S., Blouquit Y., Duchambon P., Durussel I., Cox J. A., Craescu C. T. (2003). Biochemistry.

[cit10] Veeraraghavan S., Fagan P. A., Hu H., Lee V., Harper J. F., Huang B., Chazin W. J. (2002). J. Biol. Chem..

[cit11] Cox J. A., Tirone F., Durussel I., Firanescu C., Blouquit Y., Duchambon P., Craescu C. T. (2005). Biochemistry.

[cit12] Beccia M. R., Sauge-Merle S., Lemaire D., Bremond N., Pardoux R., Blangy S., Guilbaud P., Berthomieu C. (2015). J. Biol. Inorg Chem..

[cit13] Verde V. L., Trande M., Onofrio M. D., Dominici P., Astegno A. (2018). Int. J. Biol. Macromol..

[cit14] Chen L., Madura K. (2008). Mol. Cell. Biol..

[cit15] Zhang W. L., Shi E. X., Zhao Y. Q., Yang B. S. (2018). J. Inorg. Biochem..

[cit16] Gavet O., Alvarez C., Gaspar P., Bornens M. (2003). Mol. Biol. Cell.

[cit17] Pastrana-Rios B., Reyes M., Orbeta J. D., Meza V., Narvaez D., Gomez A. M., Nassif A. R., Almodovar R., Casas A. D., Robles J., Ortiz A. M., Irizarry L., Campbell M., Colon M. (2013). Biochemistry.

[cit18] Tourbez M., Firanescu C., Yang A., Unipan L., Duchambon P., Blouquit Y., Craescu C. T. (2004). J. Biol. Chem..

[cit19] Yang A., Miron S., Duchambon P., Assairi L., Blouquit Y., Craescu C. T. (2006). Biochemistry.

[cit20] Araki M., Masutani C., Takemura M., Uchida A., Sugasawa K., Kondoh J., Ohkuma Y., Hanaoka F. (2001). J. Biol. Chem..

[cit21] Fischer T., Rodriguez-Navarro S., Pereira G., Racz A., Schiebel E., Hurt E. (2004). Nat. Cell Biol..

[cit22] Giessl A., Pulvermuller A., Trojan P., Park J. H., Choe H. W., Ernst O. P., Hofmann K. P., Wolfrum U. (2004). J. Biol. Chem..

[cit23] Gonda K., Yoshida A., Oami K., Takahashi M. (2004). Biochem. Biophys. Res. Commun..

[cit24] Guerra C., Wada Y., Leick V., Bell A., Satir P. (2003). Mol. Biol. Cell.

[cit25] Nishi R., Okuda Y., Watanabe E., Mori T., Iwai S., Masutani C., Sugasawa K., Hanaoka F. (2005). Mol. Cell. Biol..

[cit26] Resendes K. K., Rasala B. A., Forbes D. J. (2008). Mol. Cell. Biol..

[cit27] You X., Guo W., Wang L., Hou Y., Zhang H., Pan Y., Han R., Huang M., Liao L., Chen Y. (2017). Cell. Signalling.

[cit28] Krasikova Y. S., Rechkunova N. I., Maltseva E. A., Craescu C. T., Petruseva I. O., Lavrik O. I. (2012). Biochemistry.

[cit29] Huang B., Liu Y., Yao H., Zhao Y. (2020). J. Biol. Macromol..

[cit30] Lutz W., Lingle W. L., McCormick D., Greenwood T. M., Salisbury J. L. (2001). J. Biol. Chem..

[cit31] Bramson H. N., Thomas N., Matsueda R., Nelson N. C., Taylor S. S., Kaiser E. (1982). J. Biol. Chem..

[cit32] Grecu D., Assairi L. (2014). FEBS Open Bio.

[cit33] Lutz W., Lingle W. L., McCormick D., Greenwood T. M., Salisbury J. L. (2001). J. Biol. Chem..

[cit34] Zhao Y. Q., Yang J., Chao J. B., Yang B. S. (2019). Int. J. Biol. Macromol..

[cit35] Zhao Y. Q., Yan J., Chao J. B., Liang A. H., Yang B. S. (2013). J. Biol. Inorg Chem..

[cit36] Zhao X., Leon I. R., Bak S., Mogensen M., Wrzesinski K., Hojlund K., Jensen O. N. (2011). Mol. Cell. Proteomics.

[cit37] Brognard J., Hunter T. (2011). Curr. Opin. Genet. Dev..

[cit38] Salminen A., Kaarniranta K., Haapasalo A., Soininen H., Hiltunen M. (2011). J. Neurochem..

[cit39] Meyn S. M., Seda C., Campbell M., Weiss K., Hu H., Pastrana-Rios B., Chazin W. J. (2006). Biochem. Biophys. Res. Commun..

[cit40] Thompson J. R., Ryan Z. C., Salisbury J. L., Kumar R. (2006). J. Biol. Chem..

[cit41] Duan L., Liu W., Wang Z. J., Liang A. H., Yang B. S. (2010). J. Biol. Inorg Chem..

[cit42] Shi E. X., Zhang W. L., Zhao Y. Q., Yang B. S. (2017). Metallomics.

[cit43] Zhao Y. Q., Guo X. J., Yang B. S. (2019). Int. J. Biol. Macromol..

[cit44] Popescu A., Miron S., Blouquit Y., Duchambon P., Christova P., Craescu C. T. (2003). J. Biol. Chem..

[cit45] Marmur J. (1961). Int. J. Mol. Epidemiol. Genet..

[cit46] Zhao Y. Q., Cui X. F., Yang B. S. (2017). RSC Adv..

[cit47] Vreven T., Pierce B. G., wang H., Weng Z. (2013). Proteins.

[cit48] Wang Z. J., Zhao Y. Q., Ren L. X., Li G. T., Liang A. H., Yang B. S. (2007). J. Photochem. Photobiol., A.

[cit49] Förster T. (1946). Naturwissenschafien.

[cit50] Du P., Yuan L., Wu H. N., Xu K. N., Yang X. G. (2009). Int. J. Pharm. Sci. Res..

[cit51] Jones S., Thornton J. M. (1996). Proc. Natl. Acad. Sci. U. S. A..

[cit52] Sheehan J. H., Bunick C. G., Hu H., Fagan P. A., Meyn S. M., Chazin W. J. (2006). J. Biol. Chem..

[cit53] Zhao Y. Q., Song L., Liang A. H., Yang B. S. (2009). J. Photochem. Photobiol., B.

[cit54] Zhang W. L., Shi E. X., Feng J. Y., Zhao Y. Q. (2017). RSC Adv..

[cit55] Fagbemi A. F., Orelli B., Scharer O. D. (2011). DNA Repair.

[cit56] Naegeli H., Sugasawa K. (2011). DNA Repair.

[cit57] Puumalainen M. R., Ruthemann P., Min J. H., Naegeli H. (2016). Cell. Mol. Life Sci..

[cit58] Korendovych I. V., Kulp D. W., Wu Y., Cheng H., Roder H., DeGrado W. F. (2011). Proc. Natl. Acad. Sci. U. S. A..

[cit59] Wong-Deyrup S. W., Prasannan C., Dupureur C. M., Franklin S. J. (2011). J. Biol. Inorg Chem..

