# Supplementary material for: Phosphorylation promotes the endonuclease-like activity of human centrin 2
Source: RSC Adv. 2022 Aug 9;12(34):21892–903. doi: 10.1039/d2ra03402f (PMC9361469; doi:10.1039/d2ra03402f)

Electronic Supplementary Material (ESI) for RSC Advances.
This journal is © The Royal Society of Chemistry 2022

Supporting information

Phosphorylation promotes the endonuclease-like activity of human centrin 2

Jing Yang, Yaqin Zhao, Binsheng Yang*

Institute of Molecular Science, Key Laboratory of Chemical Biology and Molecular Engineering of Ministry of Education, Shanxi University, Taiyuan 030006, China

*To whom correspondence should be addressed. Tel: +86 351 7016358. Fax: +86 351 7016358. E-mail: yangbs@sxu.edu.cn

Fig.S1 Tb^{3+}-sensitized fluorescence spectra of HsCen2p with increasing concentration of Tb^{3+} in 10 mM Hepes at pH 7.4.

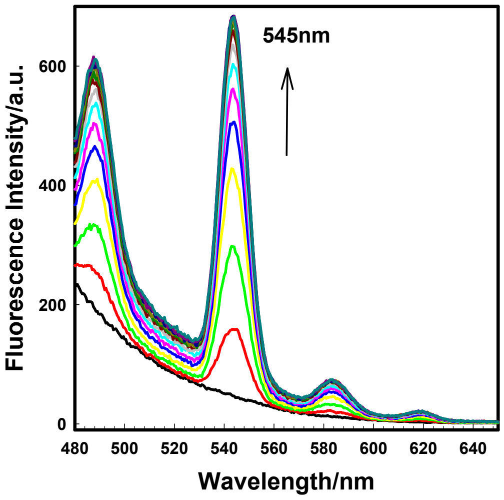

Fig.S2 (A) Resonance light scattering (RLS) spectra of HsCen2p with increasing Tb^{3+} in 10 mM Hepes at pH 7.4. (B) The best fits of log[F_i−F_0]/[F_∞−F_i] with log[Tb^{3+}]$_f$ for HsCen2 (a) and HsCen2p (b), respectively.

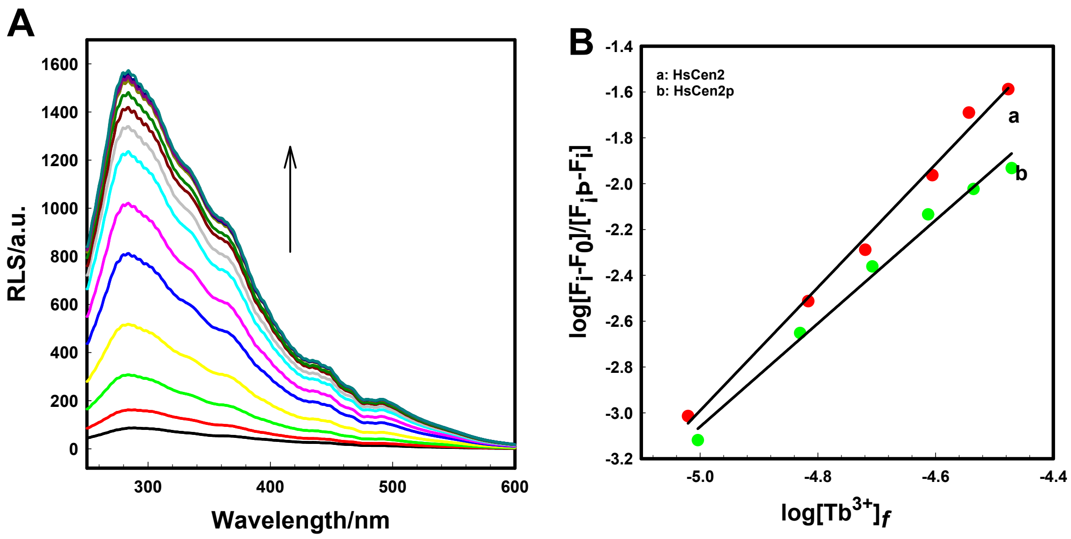

Fig.S3 Fluorescence spectra of Tb_2-HsCen2 (A) or Tb_2-HsCen2p (B) with increasing concentration of Tb^{3+} in 10 mM Hepes at pH 7.4. Inset, the titrating curves of Tb_2-HsCen2 or Tb_2-HsCen2p at 306 nm against [DNA]/[protein].

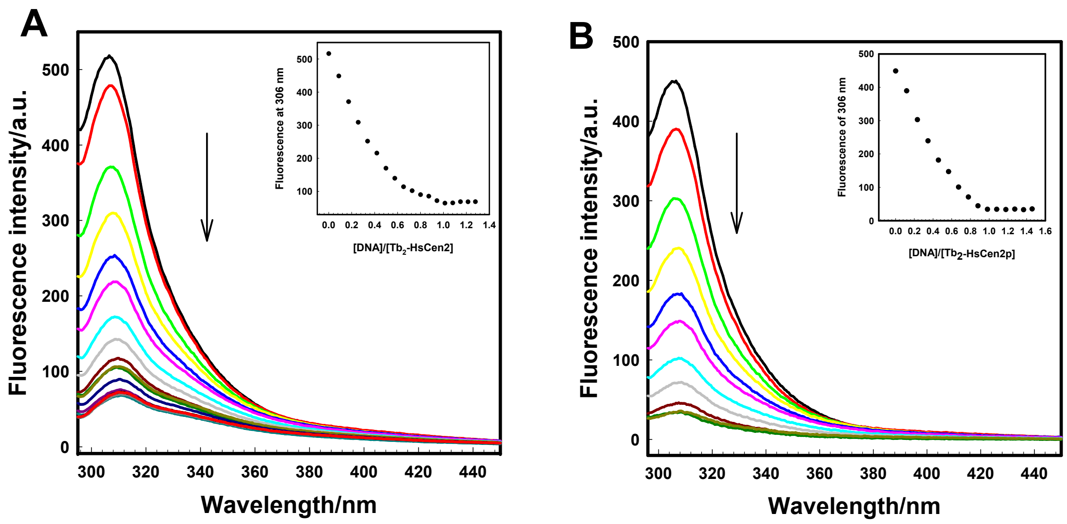

Supplement: RA-012-D2RA03402F-s001 [file RA-012-D2RA03402F-s001.pdf]
